# Rotational Thromboelastometric Profile in Early Sepsis: A Prospective Cohort Study

**DOI:** 10.3390/biomedicines12081880

**Published:** 2024-08-17

**Authors:** Piotr F. Czempik, Agnieszka Wiórek

**Affiliations:** 1 Department of Anesthesiology and Intensive Care, Faculty of Medical Sciences in Katowice, Medical University of Silesia, 40-752 Katowice, Poland; 2 Transfusion Committee, University Clinical Center of Medical University of Silesia, 40-752 Katowice, Poland

**Keywords:** coagulation, fibrinolysis, hemostasis, intensive care unit, mortality, sepsis, rotational thromboelastometry

## Abstract

Background: Coagulation abnormalities are common in sepsis patients and are associated with increased mortality. This study aimed to assess the hemostatic profile of sepsis patients using rotational thromboelastometry (ROTEM) and to find the ROTEM parameters best predicting short-term mortality. Methods: We conducted a prospective analysis of consecutive sepsis patients hospitalized in the intensive care unit. The inclusion criteria were diagnosis of sepsis or septic shock and pro-calcitonin concentration >0.5 ng mL^−1^. Clinical, standard laboratory, and ROTEM analyses were performed. Results: The study group comprised 38 (49%) males and 40 (51%) females. Median Sequential Organ Failure Assessment (SOFA) score was 8 (interquartile range IQR 5–11) points. The most common primary sites of infection were pneumonia (*n* = 27/35%), intra-abdominal (*n* = 27/35%), urinary tract infection (n=20/26%), and others (*n* = 4/6%). The following parameters evaluating fibrinogen function were outside the reference range: clotting time (CT), clot amplitude (A) at 10 and 20 min, and maximal clot firmness (MCF). Out of 78 patients, 28 (36%) died in the intensive care unit. Significant differences between survivors and non-survivors of sepsis were present for the ROTEM parameters assessing fibrinolytic activity. Conclusions: ROTEM in the early phase of sepsis reveals increased coagulation mediated through the function of fibrinogen. Non-survivors showed slightly lower fibrinolytic activity than survivors; however, it was still within test reference values. The highest predicting value was obtained by a model incorporating, among others, extrinsic coagulation pathway fibrinolytic parameters.

## 1. Introduction

Sepsis is defined as “life-threatening organ dysfunction caused by a dysregulated host response to infection” [1]. Several diagnostic and prognostic tools have been studied over the years in sepsis patients. The most established tool for diagnosis and prognostication in sepsis is the Sequential Organ Failure Assessment (SOFA) score, proposed by the European Society of Intensive Care Medicine (ESICM) during the consensus meeting in Paris in 1994 and officially published in 1996. It comprises six sub-scores for major organ dysfunctions: cardiovascular, respiratory, hepatic, renal, neurological, and coagulation [2]. For each organ system, a single vital or laboratory parameter was chosen that reflects the degree of organ loss of function, defining the severity of ongoing sepsis and predicting mortality risk. Coagulation abnormalities are common in sepsis patients. For assessment of coagulation in the SOFA score, platelet count (PLT) was selected as a biomarker of sepsis, with 1 point given for the PLT range 100–149 × 10^3^ µL^−1^, 2 points for the PLT range 50–99 × 10^3^ µL^−1^, 3 points for the 20–50 × 10^3^ µL^−1^ PLT range, and finally 4 points for PLTs <20 × 10^3^ µL^−1^ [3]. Improved understanding of the pathophysiology of sepsis through novel methods for measuring and predicting the progression of the disease can improve sepsis patients’ outcomes.

Coagulation is a complex process that depends upon three main coagulation phases: primary hemostasis (platelet plug formation), secondary hemostasis (fibrin formation), and fibrinolysis [4]. Sepsis is a condition that can affect different coagulation phases. Coagulopathy in sepsis patients is associated with increased mortality [5]. Distinctions have been made between early-phase sepsis-induced coagulopathy (SIC) and late-phase sepsis-associated disseminated intravascular coagulation (DIC) [6]. Diagnostic criteria for these two entities have been based on standard laboratory tests (SLTs) for coagulation. However, SLTs for coagulation have relevant limitations. Assessment of hemostasis in sepsis patients is a difficult task, as sepsis affects a delicate balance between activation and inhibition of coagulation, and fibrinolysis. The thrombin generation test can be used to assess the activation of the coagulation system; however, this test is not widely available and is mostly used as a research tool. Inhibition of coagulation can be assessed by measuring activities of natural anticoagulants like antithrombin (AT), protein C, protein S, and tissue factor pathway inhibitor; yet again, these are not the tests (perhaps except for AT) that are commonly at the disposal of a clinician. Although increased fibrinolytic activity can be indirectly judged by increased concentration of D-dimers (DDs) or other fibrin degradation products (rarely available), these tests lack specificity [7]. Viscoelastic point-of-care tests (POCTs) of coagulation, rotational thromboelastometry (ROTEM^®^) or rotational thromboelastography (TEG^®^), are significant developments in the modern armamentarium for hemostasis assessment. An essential advantage of viscoelastic tests is the ability to assess coagulation and fibrinolysis directly. The advantages of viscoelastic tests compared to standard laboratory coagulation testing are manifold. Firstly, there is a short turnaround time to obtain the results compared to laboratory testing, for some parameters as short as 2 min, which is ideal for emergency situations. Secondly, clot formation is visualized in real-time at the point of care in the form of so-called temograms. Thirdly, testing is performed on a whole blood sample, not only plasma, as in standard laboratory tests of coagulation, allowing for analyzing the interaction between clotting factors, platelets, and red blood cells [8].

The aims of this study were to assess the hemostatic profile of sepsis patients using ROTEM and to find ROTEM parameters that best predicted short-term mortality. 

## 2. Materials and Methods

**Figure 1:**
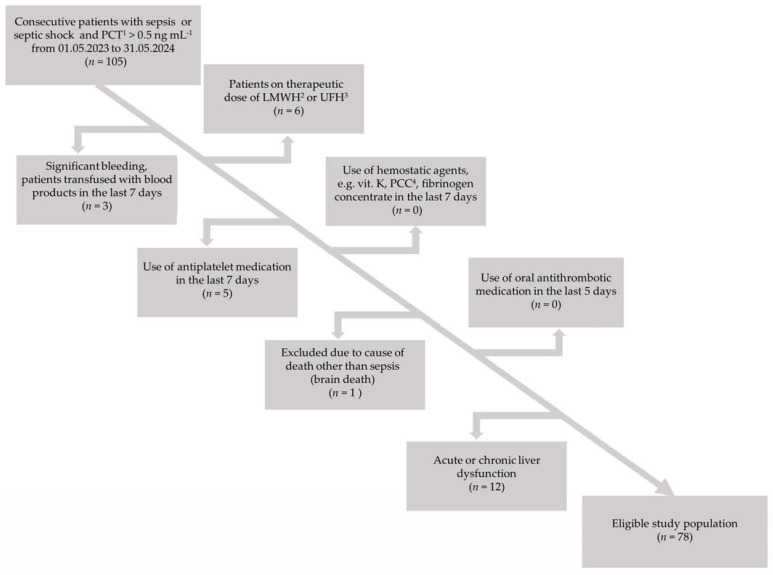
The study flow chart. ^1^ Procalcytonin. ^2^ Low-molecular-weight heparin. ^3^ Unfractionated heparin. ^4^ Prothrombin complex concentrate.

We conducted a prospective analysis of consecutive sepsis patients hospitalized in a 10-bed mixed medical–surgical intensive care unit (ICU) in the University Clinical Center of Medical University of Silesia in Katowice, Poland (663 hospital beds, 2017 employees, 45351 hospitalizations annually). The enrollment period was from May 2023 to May 2024. The inclusion criteria were diagnosis of sepsis or septic shock and procalcitonin (PCT) concentration >0.5 ng mL^−1^. Sepsis and septic shock were diagnosed using the third international definition [1]. Although PCT determination is not required for the diagnosis of sepsis or septic shock according to the most recent Surviving Sepsis Campaign guidelines [9], it may be difficult in the ICU to distinguish acute organ dysfunction caused by sepsis from organ dysfunction due to other causes. Therefore, we decided to use PCT concentration as an additional diagnostic criterion for sepsis and septic shock, basing our decision on the Japanese clinical practice guidelines for managing sepsis and septic shock [10]. The cut-off value for PCT was set at >0.5 ng mL^−1^ as it was proven that systemic infection is unlikely with this concentration [11]. The exclusion criteria were factors having a potential impact on hemostasis: clinically significant bleeding (World Health Organization Bleeding Grade 3 or 4) [12], use of hemostatic agents (e.g., vitamin K, prothrombin complex concentrate, fibrinogen concentrate) in the last 7 days, transfusion of blood products (e.g., fresh-frozen plasma, cryoprecipitate, platelet concentrate) in the previous 7 days, use of therapeutic doses of low-molecular-weight heparins (LMWHs), use of antiplatelet medications in the last 7 days, use of oral antithrombotic medications in the last 5 days, and acute or chronic liver dysfunction (e.g., acute hepatitis, liver cirrhosis). The study flow chart is presented in Figure 1.

### 2.1. Clinical Data

Clinical data included primary site of infection, SOFA score, time interval between ICU admission and patient enrollment (in days), type of LMWH used, time interval between LMWH administration and collection of blood (in hours), ICU length of stay (LOS), ICU mortality, body mass index (BMI), SIC score [13], and DIC score [14]. Regarding DIC criteria, cut-offs for moderate and severe increases in DD concentrations were set at 2400 and 22000 ng mL^−1^, respectively [15].

### 2.2. Laboratory Data

No extra blood was needed to perform POCT for coagulation or SLTs for coagulation because it is obligatory in the local ICU to determine SLTs for coagulation in sepsis patients. The same test tube was used for both types of tests. It is also necessary to determine the complete blood count (CBC) and biochemistry panel for sepsis patients. All of these laboratory parameters are necessary to calculate the initial SOFA score and monitor it during treatment. A full laboratory diagnostic panel in sepsis patients requires patient blood to be collected in 3 different test tubes: an ethylenediaminetetraacetic acid (EDTA) test tube (2 mL), a 3.2% sodium citrate test tube (2 mL), and a clot activator test tube (2.5 mL). Blood was withdrawn through an arterial cannula using a vacuum system (BD Vacutainer^®^, Franklin Lakes, NJ, USA). Every effort was taken not to contaminate blood samples with unfractionated heparin (UFH) present in the arterial line flushing solution. Serum biochemical parameters included PCT, C-reactive protein (CRP), creatinine, urea, blood urea nitrogen (BUN), and total bilirubin. Standard laboratory tests of coagulation included fibrinogen concentration, DD, activated partial thromboplastin time (aPTT), prothrombin time (PT), international normalized ratio (INR), thrombin time (TT), CBC with differential, reticulocyte count with reticulocyte subpopulations, reticulocyte maturity index (RMI), and reticulocyte hemoglobin equivalent (Ret-He). Fibrinogen concentration was measured using the Clauss method, using thrombin to measure fibrinogen in human-citrated plasma on the IL Coagulation System [16]. 

#### Rotational Thromboelastometric Parameters

Rotational thromboelastometry was performed using a ROTEM delta analyzer (Tem Innovations GmbH, Munich, Germany) according to instructions provided by the manufacturer. Analyses were conducted by the authors who are experienced in running the test. Assays were run for the entire 60 min period. Assays were started immediately after blood sampling to minimize the risk of pre-analytical error. The same test tube was used to run the ROTEM and determine SLTs for coagulation. First, blood was withdrawn from a test tube with a semi-automatic pipette to run the ROTEM. Then, the test tube with the remaining blood was sent to the Central Laboratory for further analysis. The amount of remaining blood was adequate (approximately 0.8 mL) to determine SLTs for coagulation. A written comment was sent to the laboratory diagnosticians that the ratio of blood to anticoagulant in the remaining blood was correct. Four ROTEM assays were run simultaneously: INTEM, EXTEM, FIBTEM, and APTEM. The parameters of interest measured in the four assays were clotting time (CT), clot formation time (CFT), alpha angle (AA), clot amplitude at different time points (minutes) (A10, A20), maximum clot firmness (MCF), maximum lysis (ML), lysis index at 30 min (LI30), and lysis index at 45 min (LI45). The intrinsic coagulation pathway (INTEM) reagent contains ellagic acid as the contact activator, leading to activation of the intrinsic coagulation pathway and estimating the function of the coagulation factors XII, XI, IX, VIII, factors of the common coagulation pathway. The extrinsic pathway (EXTEM) reagent includes the tissue factor leading to the initiation of the extrinsic coagulation pathway and assesses the function of factor VII and common coagulation pathway factors. The fibrinogen (FIBTEM) reagent combines the EXTEM base for clot assessment after adding cytochalasin D, a platelet inhibitor, allowing for the isolated appraisal of the fibrin component of the clot. The APTEM reagent contains aprotinin, an anti-fibrinolytic agent, that confirms or excludes hyperfibrinolysis.

### 2.3. Statistical Analysis

Statistical analysis was performed using licensed statistical software (MedCalc v.18, Ostend, Belgium). Quantitative variables were expressed as medians and interquartile ranges (IQR, i.e., 25pc–75pc). The Shapiro–Wilk test was used to verify the type of distribution of quantitative variables. Qualitative variables were expressed as frequencies and percentages. Between-group differences for quantitative variables were calculated with the Kruskal–Wallis test or independent samples t-test, depending on the type of distribution. The Chi-squared test was applied for categorical variables, with Fisher’s exact test adjustment when applicable. Receiver operating characteristic (ROC) curves were drawn, and the area under the ROC curves (AUROC) was calculated to determine the predictive value of analyzed parameters and the outcome. The ROC analysis was also performed to assess the optimal cut-off values in outcome prediction statistically. All tests were two-sided. *P*-values were given with accuracy to three decimal places. A *p*-value <0.05 was considered statistically significant.

## 3. Results

Most patients were enrolled in the study at the moment of ICU admission or the next day. The median time interval between ICU admission and blood collection was 1 day (IQR 0–2). The study group comprised 78 patients: 38 (49%) males and 40 (51%) females. The median age in the study group was 65 (IQR 52–71) years. The majority of the study subjects (*n* = 49, 62.8%) were not receiving LMWH before enrollment in the study; 23 (29.5%) were receiving a prophylactic dose (40 mg once daily) of enoxaparin (Clexane, Sanofi-Aventis, Frankfurt, Germany) and 6 (7.7%) a prophylactic dose (5000 units once daily) of dalteparin (Fragmin, Pfizer, Puurs, Belgium). The median interval between LMWH subcutaneous injection and collection of blood was 24 (IQR 12.5–24.7) hours. There were seven (8.9%) study subjects in whom SIC was diagnosed (unable to calculate score in one study subject). The median SIC score in the study group was 3 (IQR 2–4) points (diagnosis of SIC at 5 points). There was one (1.3%) study subject in whom DIC was diagnosed (unable to calculate the score in two study subjects). The median DIC score in the study group was 2 (IQR 0–3) points (diagnosis of DIC at 5 points). The median SOFA score was 8 (IQR 5–11) points. The most common primary sites of infection were pneumonia (*n* = 27/35%), intra-abdominal (*n* = 27/35%), urinary tract infection (*n* = 20/26%), and others (*n* = 4/6%), i.e., cholangitis, meningitis, intrauterine, unspecified. The median BMI in the study group was 25 (IQR 24–31) kg m^−2^. Out of 78 patients, 28 (36%) died in the ICU. There were no significant differences in ICU mortality in patients with different primary sites of infection (*p* = 0.194). There was a significant difference (*p* < 0.001) in the median SOFA score between survivors (7, IQR 4–9 points) and non-survivors (11, IQR 8–12 points). The median ICU LOS was 9 (IQR 5–20) days.

The laboratory parameters for the study group are presented in Table 1. 

The coagulation parameters that were outside the reference ranges were PT (prolonged), fibrinogen concentration (increased), and DD (increased).

The ROTEM parameters for the study population are presented in Table 2. Among the measurements of viscoelastic essays, the following FIBTEM parameters were outside the reference range: CT, A10, A20, and MCF.

Out of 78 patients, 28 (36%) died in the ICU. The differences in thromboelastometric parameters between survivors and non-survivors are shown in Table 3. 

Statistically significant differences between survivors and non-survivors of sepsis were present for the ROTEM parameters assessing fibrinolytic activity: LI30 (INTEM, APTEM), LI45 (INTEM, EXTEM, APTEM), and ML (INTEM, EXTEM, FIBTEM, APTEM). These parameters suggested slightly lower fibrinolytic activity among non-survivors. However, all these parameters were within the reference ranges for survivors and non-survivors of sepsis.

In the AUROC analysis for mortality prediction, the strongest predictors were as follows, in decreasing order: INTEM ML, INTEM LI45, EXTEM ML, and APTEM ML (Table 4).

In the logistic regression model for short-term mortality prediction, ROTEM parameters with significant AUROC values (Table 4), adjusted for sex, age, primary site of infection, and SOFA score, revealed that EXTEM ML and EXTEM LI45 are the most valuable predictors, with p=0.028 and p=0.029, respectively. The overall fitness of the model was excellent, with an AUROC of 0.913 (*p* < 0.001).

## 4. Discussion

This prospective study aimed to characterize the hemostatic profile of sepsis patients using ROTEM and compare ROTEM parameters in survivors and non-survivors of sepsis (ICU mortality). 

ROTEM revealed abnormalities in FIBTEM: prolonged CT and increased clot amplitude at different time points. Increased amplitudes in FIBTEM suggest increased coagulation mediated through fibrinogen function. Fibrinogen maintains the highest concentration among the plasma clotting factors. The reference range value for fibrinogen concentration is approximately 200–400 mg dL^−1.^ Fibrinogen is also an acute-phase reactant; therefore, its concentration increases during systemic inflammatory conditions like sepsis or septic shock [17]. 

Coagulopathy in sepsis may take the form of SIC (early stage) or sepsis-associated DIC (late stage) [13]. The diagnostic criteria for sepsis-induced coagulopathy are as follows: SOFA score, PLT, and INR [13]. A patient may receive 0–2 points for every SIC criterion depending on the degree of abnormalities. A minimum of 5 points is required to confirm SIC. In our study group, the patients generally scored 2 points for SOFA, 0 points for PLT, and 0 points for INR, so the diagnosis of SIC could not be confirmed. However, our study subjects presented with certain features of SIC. Both SLTs and ROTEM FIBTEM confirmed increased fibrinogen concentration and function, staying in line with the features of SIC [18]. However, another feature of SIC—impaired fibrinolysis due to increased concentration of plasminogen activator inhibitor-1 (PAI-1)—was not met in our total study population [19]. Increasing PAI-1 in sepsis patients leads to the shutdown of fibrinolysis, which correlates with the severity of coagulopathy [20]. The reason for normal fibrinolytic activity in our entire patient population could be because patients were in the early phase of sepsis, and abnormalities in fibrinolysis did not have time to develop fully. Moreover, a blood sample for each patient was drawn only once. 

A study by Scarlatescu et al. compared sepsis survivors and non-survivors with healthy controls, focusing particularly on ROTEM lysis parameters. The study revealed observations similar to our own regarding the lytic resistance recorded in sepsis patients. Among the studied ROTEM parameters, the authors showed the tendency for slower clotting initiation, reflected especially in the EXTEM CT in the sepsis patients’ population compared to the healthy control group. Also, the results pointed towards a lower grade of clot degradation measured as lysis index at 45 and 60 min of ROTEM measurement, with a notable number of patients having 0% lysis at 45 min, especially in the non-survivor subgroup. As means considered for patient prognostication, most ROTEM parameters studied between healthy controls and sepsis non-survivors differed significantly. However, when healthy controls were compared with sepsis survivors, only the lysis indexes at 45 and 60 min were different [21]. 

The International Society on Thrombosis and Hemostasis (ISTH) defines DIC as “an acquired syndrome characterized by the intravascular activation of coagulation with loss of localization arising from different causes. It can originate from and cause damage to the microvasculature, which, if sufficiently severe, can produce organ dysfunction” [14]. There are several sub-types of DIC, which can be classified as suppressed fibrinolysis-type, enhanced fibrinolysis-type, and balanced fibrinolysis-type [22]. Sepsis-associated DIC represents the suppressed fibrinolysis type, which translates into insufficient fibrinolysis due to highly activated coagulation relative to mildly elevated fibrinolysis, resulting in organ dysfunction caused by microcirculatory clot formation rather than bleeding [23]. The diagnostic criteria for DIC diagnosis include PLT, DD, fibrinogen concentration, and PT. In our study subjects, only one was confirmed to have sepsis-associated DIC. 

The aspect of clotting activation, alongside generalized pro-inflammatory mobilization, is an essential element of host defense during the systemic reaction to sepsis. Cytokines, such as tumor necrosis factor (TNF) α and interleukin (IL) 1, 2, 6, and 8, among others, cause neutrophil endothelial cell adhesion, activate the complement, and initiate coagulation, leading to the generation of microthrombi [24].

Coagulation abnormalities are widely manifested among sepsis patient populations, regardless of the infection point of origin [25]. Some forms of coagulopathies may only be detected by sensitive markers of clotting factor activation; some manifest through a more detectable decrease in platelet count and subclinical or an overt prolongation of global clotting times to fulminant DIC characterized by simultaneous widespread microvascular thrombosis and profuse bleeding from various sites [26].

Current research recognizes platelet count and platelet reactivity as valid mediators of inflammatory response in sepsis. This occurs through interactions with immune cells’ secretion of immunomodulators and the formation of platelet–platelet and platelet–leukocyte aggregates [27]. Platelet aggregation has been shown to increase in an uncomplicated course of sepsis; however, platelet count decreased over time in patients who developed severe sepsis [28]. This aligns with our findings of significantly lower platelet count in the non-survivors subgroup, which scored higher on the SOFA score. This, however, was not mirrored in the ROTEM parameters associated with platelet availability, such as clot amplitudes in the EXTEM assay, which were recorded closer to the upper reference values, contributing to a hypercoagulable state [29].

Our study has certain limitations. Firstly, it is a single-center study, and our sepsis population may not reflect sepsis patients in other institutions, especially regarding the primary site of infection. Secondly, there were some exclusions; in particular, patients receiving therapeutic anticoagulation were not included. However, it is not possible to accurately assess hemostasis in these patients. Thirdly, in our study, we used a semi-automatic ROTEM Delta analyzer that requires mixing reactants with blood. However, the analyzer was operated by clinicians who were experienced in this method, strictly following the manufacturer’s instructions. A fully automated ROTEM analyzer is available on the market. We do not know if utilizing an improved version of the ROTEM analyzer would improve the accuracy of determination.

The presented research is a preliminary cohort study comprising 78 patients. Therefore, further research is required to gain full insight into the research topic.

## 5. Conclusions

Rotational thromboelastometry in the early phase of sepsis reveals increased coagulation mediated through the function of fibrinogen. Non-survivors showed slightly lower fibrinolytic activity compared to survivors; however, it was still within test reference values. The highest mortality-predicting value was obtained by a model that incorporated extrinsic coagulation pathway fibrinolytic parameters apart from standard sepsis mortality predictors. Hemostasis abnormalities in sepsis are dynamic. Rotational thromboelastometry captures these dynamic changes and can assist clinicians in deciding on the most appropriate coagulation management approach. 

## Figures and Tables

**Table 1:** Laboratory parameters in the study group.

Parameter	Median Value, IQR ^1^	Reference Range
Procalcitonin [ng mL^−1^]	3.51 (1.12–18.30)	<0.5
C-reactive protein [mg L^−1^]	176 (117–286)	<5.0
Creatinine [mg dL^−1^]	1.12 (0.67–1.60)	0.67–1.17
Estimated GFR ^2^ [mL min^−1^]	60 (37–60)	>60
Blood urea nitrogen [mg dL^−1^]	28.99 (17.69–42.81)	7.90–20.00
Urea [mg dL^−1^]	61.3 (38.8–91.5)	17.1–49.2
Bilirubin [mg dL-^−1^]	0.60 (0.31–0.86)	0.30–1.00
White blood cells [× 10^3^ µL^−1^]	11.1 (7.1–17.9)	4.0-10.0
Hemoglobin [g L^−1^]	106.0 (85.0–123.0)	135.0–165.0
Hematocrit [%]	31.9 (26.4–35.6)	40–53
Platelets [× 10^3^ µL^−1^]	213 (159–283)	130-400
**Prothrombin time [s]**	**13.9 (12.8–16.4)**	**9.4–12.5**
International normalized ratio	1.15 (1.06–1.37)	0.80–1.20
**Quick index [%]**	**76.0 (58.8–86.0)**	**80–120**
Activated partial thromboplastin time [s]	32.5 (30.3–37.3)	25.4–36.9
Thrombin time [s]	15.7 (14.3–16.8)	10.3–16.6
**Fibrinogen [mg dL^−1^]**	**523 (389–640)**	**200–393**
**D-dimers [ng mL^−1^]**	**2613 (1716–5651)**	**<500**

^1^ Interquartile range. ^2^ Glomerular filtration rate according to Modification of Diet in Renal Disease. **In bold: abnormal results.**

**Table 2:** Rotational thromboelastometric parameters in the study group.

Parameter	Median Value, IQR ^1^	Reference Range
INTEM CT ^2^ [s]	195 (177–211)	100–240
INTEM CFT ^3^ [s]	59 (49–70)	30–110
INTEM alpha angle [°]	78 (76–80)	70–83
INTEM A10 ^4^ [mm]	65 (60–69)	44–66
INTEM A20 ^5^ [mm]	70 (65–74)	50–71
INTEM MCF ^6^ [mm]	70 (66–75)	50–72
INTEM LI30 ^7^ [%]	100 (99–100)	94–100
INTEM LI45 ^8^ [%]	97 (95–99)	-
INTEM ML ^9^ [%]	5 (2–8)	0–15
EXTEM CT [s]	77 (69–88)	38–79
EXTEM CFT [s]	59 (48–70)	34–159
EXTEM alpha angle [°]	78 (77–80)	63–83
EXTEM A10 [mm]	65 (60–71)	43–65
EXTEM A20 [mm]	69 (65–73)	50–71
EXTEM MCF [mm]	70 (67–74)	50–72
EXTEM LI30 [%]	99 (98–100)	94–100
EXTEM LI45 [%]	96 (91–98)	-
EXTEM ML [%]	8 (4–14)	0–15
**FIBTEM CT [s]**	**73 (65–82)**	**38–62**
FIBTEM CFT [s]	114 (65–312)	-
FIBTEM alpha angle [°]	77 (74–79)	-
**FIBTEM A10 [mm]**	**24 (20–31)**	**7–23**
**FIBTEM A20 [mm]**	**25 (21–33)**	**8–24**
**FIBTEM MCF [mm]**	**26.5 (22.0–34.0)**	**9–25**
FIBTEM LI30 [%]	100 (100–100)	-
FIBTEM LI45 [%]	100 (100–100)	-
FIBTEM ML [%]	0 (0–1)	-
APTEM CT [s]	74 (68–89)	-
APTEM CFT [s]	60 (48.0–70)	-
APTEM alpha angle [°]	78 (77–80)	-
APTEM A10 [mm]	65 (60–70)	-
APTEM A20 [mm]	70 (66–74)	-
APTEM MCF [mm]	71 (67–75)	-
APTEM LI30 [%]	100 (99–100)	-
APTEM LI45 [%]	97 (94–99)	-
APTEM ML [%]	6 (3–8)	-

^1^ Interquartile range. ^2^ Clotting time. ^3^ Clot formation time. ^4^ Clot amplitude measured at 10 min. ^5^ Clot amplitude measured at 20 min. ^6^ Maximum clot firmness. ^7^ Lysis index at 30 min. ^8^ Lysis index at 45 min. ^9^ Maximum lysis. **In bold: abnormal results.**

**Table 3:** Rotational thromboelastometric parameters in survivors and non-survivors of sepsis.

Parameter	Survivors (*n* = 50/64%)	Non-Survivors (*n* = 28/36%)	*p* ^1^
INTEM CT ^2^ [s]	192 (177–207)	197 (187–229)	0.177
INTEM CFT ^3^ [s]	58 (48–70)	62 (54–71)	0.396
INTEM alpha angle [°]	79 (76–80)	78 (76–80)	0.526
INTEM A10 ^4^ [mm]	65 (61–71)	64 (59–68)	0.255
INTEM A20 ^5^ [mm]	70 (65–75)	70 (65–72)	0.327
INTEM MCF ^6^ [mm]	70 (66–75)	70 (66–73)	0.573
**INTEM LI30 ^7^ [%]**	**99 (99–100)**	**100 (100–100)**	**0.007**
**INTEM LI45 ^8^ [%]**	**96 (94–98)**	**99 (96–100)**	**<0.001**
**INTEM ML ^9^ [%]**	**6 (4–8)**	**2 (1–6)**	**<0.001**
EXTEM CT [s]	76 (70–86)	77 (68–91)	0.971
EXTEM CFT [s]	58 (48–68)	64 (51–75)	0.193
EXTEM alpha angle [°]	79 (77–80)	78 (76–80)	0.132
EXTEM A10 [mm]	66 (61–72)	64 (59–68)	0.139
EXTEM A20 [mm]	70 (65–74)	68 (63–72)	0.172
EXTEM MCF [mm]	71 (67–75)	69 (67–73)	0.169
EXTEM LI30 [%]	99 (96–100)	100 (99–100)	0.062
**EXTEM LI45 [%]**	**95 (90–97)**	**97 (95–100)**	**0.003**
**EXTEM ML [%]**	**10 (6–17)**	**5 (2–8)**	**<0.001**
FIBTEM CT [s]	72 (65–80)	75 (66–85)	0.283
FIBTEM CFT [s]	114 (66–312)	117 (61–298)	0.923
FIBTEM alpha angle [°]	77 (74–78)	77 (74–80)	0.279
FIBTEM A10 [mm]	24 (19–31)	23 (21–30)	0.736
FIBTEM A20 [mm]	26 (20–34)	25 (23–31)	0.661
FIBTEM MCF [mm]	26 (20–34)	27 (24–34)	0.417
FIBTEM LI30 [%]	100 (100–100)	100 (100–100)	0.367
FIBTEM LI45 [%]	100 (100–100)	100 (100–100)	0.073
**FIBTEM ML [%]**	**0 (0–2)**	**0 (0–0)**	**0.045**
APTEM CT [s]	73 (65–88)	77 (69–91)	0.336
APTEM CFT [s]	56 (48–69)	62 (53–74)	0.177
APTEM alpha angle [°]	79 (77–81)	78 (76–80)	0.169
APTEM A10 [mm]	66 (60–72)	64 (59–69)	0.307
APTEM A20 [mm]	70 (66–75)	70 (65–74)	0.386
APTEM MCF [mm]	71 (67–76)	70 (67–74)	0.539
**APTEM LI30 [%]**	**100 (99–100)**	**100 (100–100)**	**0.014**
**APTEM LI45 [%]**	**96 (94–97)**	**98 (97–100)**	**0.004**
**APTEM ML [%]**	**7 (5–9)**	**3 (1–6)**	**0.001**

^1^ *P*-value for inter-group differences calculated with independent samples t-test or Kruskal–Wallis test. ^2^ Clotting time. ^3^ Clot formation time. ^4^ Clot amplitude measured at 10 min. ^5^ Clot amplitude measured at 20 min. ^6^ Maximum clot firmness. ^7^ Lysis index at 30 min. ^8^ Lysis index at 45 min. ^9^ Maximum lysis values are medians and interquartile ranges (IQR). **In bold: statistically significant differences.**

**Table 4:** Rotational thromboelastometric parameters in prediction of short-term mortality in sepsis patients.

Parameter	AUROC ^1^ (95% CI ^2^)	Cut-Off Value	*p* ^3^
INTEM LI30 ^4^ [%]	0.665 (0.548–0.769)	>99	0.002
INTEM LI45 ^5^ [%]	0.735 (0.622–0.829)	>98	<0.001
INTEM ML ^6^ [%]	0.744 (0.633–0.836)	≤2	<0.001
EXTEM LI45 [%]	0.707 (0.592–0.805)	>94	0.002
EXTEM ML [%]	0.729 (0.617–0.824)	≤7	<0.001
FIBTEM ML [%]	0.610 (0.492–0.720)	≤1	0.025
APTEM LI30 [%]	0.642 (0.524–0.749)	>99	0.005
APTEM LI45 [%]	0.702 (0.585–0.802)	>97	0.002
APTEM ML [%]	0.724 (0.611–0.820)	≤2	<0.001

^1^ Area under receiver operating characteristic curve. ^2^ Confidence interval. ^3^ *P*-value. ^4^ Lysis index at 30 min. ^5^ Lysis index at 45 min. ^6^ Maximum lysis.

## Data Availability

The raw data supporting the conclusions of this article will be made available by the authors upon request.
